# Medium Effects on the Dissociation of Weak Acids in Methanol-Water Solvents

**DOI:** 10.6028/jres.068A.028

**Published:** 1964-06-01

**Authors:** E. E. Sager, R. A. Robinson, R. G. Bates

## Abstract

A spectrophotometric method has been used to determine the dissociation constants of *o*-chloroanilinium ion, *m*-nitroanilinium ion, and 4-chloro-2,6-dinitrophenol in methanol- water solvents at 25 °C. The ranges of solvent compositions (in weight percent methanol) were as follows: *o*-chloroanilinium ion, 0 to 99.9; *m*-nitroanilinium ion, 0 to 93.7; and 4-chloro-2,6-dinitrophenol, 0 to 33.4. The *pK* of the first two acids falls with addition of methanol and passes through a minimum when the mole fraction of methanol in the solvent mixture is about 0.7. The *pK* of the substituted phenol, however, rises as the dielectric constant of the solvent is decreased by addition of methanol. It is suggested that the total medium effect for both types of acid can be explained by the superposition of an electrostatic effect and a nonelectrostatic effect. The latter is a constant for each particular solvent composition and probably characterizes the acid-base property of the medium itself.

## 1. Introduction

The nature of the parameters that describe the effect of a changing medium on the free energy of electrolytes is not yet well understood. There is considerable agreement, however, that two effects, one electrostatic and the other chemical, can be identified [1].^1^ The electrostatic effect is more easily estimated than is the chemical term. The Born equation [2] furnishes an estimate of the former by contrasting the work required to charge spherical ions in the two media of different dielectric constant. This simple approach ignores specific chemical effects and second-order interactions and treats the medium as a dielectric continuum. Nevertheless, it may furnish estimates of medium effects that are completely reasonable and, when a comparison with experiment is possible, also qualitatively correct.

The effects of changes in the composition of a solution on an acid-base equilibrium in that solution

$$A⇋B+H^{+}$$ (1)

are embodied in the activity coefficients, *γ_i_*, which are part of the thermodynamic formulation of the expression for the equilibrium constant *K*, in this special case the acidic dissociation constant of A. On the molal (*m*) scale,

$$K=\frac{m_{H}m_{B}}{m_{A}}\frac{\gamma_{H}\gamma_{B}}{\gamma_{A}}.$$ (2)

The numerical value of *K* is fixed by choice of a standard state in which the activity coefficients are assigned values of unity. In aqueous solutions, the customary standard state is chosen so that *γ_i_* approaches unity as *m* approaches 0.

When the composition of the solvent medium changes as well as the solute concentration (and ionic strength), it is convenient to separate each activity coefficient, *γ_i_* into two factors,

$$\gamma_{i}\equiv {\;}_{m}\gamma_{i}\cdot_{s}\;\gamma_{i}\cdot$$ (3)

*γ_i_* is measured relative to the standard state in pure water and becomes unity only in an infinitely dilute *aqueous* solution. On the contrary, the activity coefficient ${\;}_{s}\;\gamma_{i}$ in eq (3) becomes unity when *m*=0 in the solvent s, whereas ${\;}_{m}\;\gamma_{i}$ has a value different from unity whenever the solvent differs from pure water.

The “salt effect” ${\;}_{s}\;\gamma_{i}$ varies with the solute concentration, but the “medium effect” ${\;}_{m}\;\gamma_{i}$ is a function only of the free energy of the species *i* in the two standard states (namely those in the medium s and in water). When ionic species are involved, ${\;}_{s}\;\gamma_{i}$ can be estimated by the Debye-Hückel equation with appropriate allowance for the effect of altering the dielectric constant of the medium s on the interionic forces.

The activity coefficient ${\;}_{m}\;\gamma_{i}$ measures the difference in the standard free energy of *i* in the two media:

$$\Delta G_{i}^{0}={\;}_{s}G_{i}^{0}-{\;}_{w}G_{i}^{0}=RT\ln{\;}_{m}\;\gamma_{i}.$$ (4)

Electrostatic considerations show that there will be a change of free energy when free ions are transferred from one medium to another of different dielectric constant, even though the ions remain free in the second medium also. Yet chemical interactions, of which solvation is the most important, must surely influence the medium effect as well. For this reason, the nonelectrostatic contribution to the free energy may change considerably as the solvent composition is altered.

The reaction of the solvent with the acid A and the base B in eq (1) (solvation) is a manifestation of the acid-base property of the solvent. Hence, the electrostatic treatment accounts satisfactorily for the change of the equilibrium constant of eq (1) with dielectric constant only when the change of dielectric constant is accomplished without a change in the acid-base properties of the solvent. This condition is evidently difficult to achieve, but the essential correctness of these concepts is attested by the observation that the ratios of the dissociation constants of different acids A to that of a chosen reference acid of the same charge type are often nearly independent of the dielectric constant of the solvent.

In view of the complexity of the medium effect on acid-base dissociation, it is not surprising that the Born electrostatic treatment alone is incapable of yielding useful quantitative data. It is indeed rather remarkable that the predictions of the Born equation are so often qualitatively correct. Considering only the electrostatic contribution to the free energy, one can write, by combining the Born equation with eq (4),

$$\ln{\;}_{m}\;\gamma_{i}=\frac{Nz_{i}^{2}e^{2}}{2RTr}\left(\frac{1}{\epsilon_{s}}-\frac{1}{\epsilon_{w}}\right),$$ (5)

where *N* is the Avogadro number, *z_i_* is the number of charges borne by the species *i, e* is the electronic charge, *r* is the radius of the (spherical) ion or molecule *i, ϵ*_s_ and *ϵ*_w_ are the dielectric constants of the two solvent media, *R* is the gas constant, and *T* is the temperature in degrees Kelvin. The mass law expression, eq (2), for equilibrium (1) yields

$${\;}_{s}K={\;}_{w}K\cdot \frac{{\;}_{m}\gamma_{A}}{{\;}_{m}\gamma_{H}\cdot {\;}_{m}\gamma_{B}},$$ (6)

where _w_*K* and _s_*K* are the dissociation constants referred to the standard states in water and in solvent s, respectively.

By combination with eq (5) we obtain

$$p\left({\;}_{s}K\right)-p\left({\;}_{w}K\right)=\frac{Ne^{2}}{4.6052RT}\left(\frac{1}{\epsilon_{s}}-\frac{1}{\epsilon_{w}}\right)\left(\frac{z_{B}^{2}}{r_{B}}-\frac{z_{A}^{2}}{r_{A}}+\frac{1}{r_{H}}\right).$$ (7)

When the dissociation equilibrium (1) is of the charge type A^0^B^−^ (for example, A^0^= uncharged acetic acid, B^−^= acetate anion; *z_A_*=0), eq (7) predicts that *p*(_s_*K*) will be greater than *p*(_w_*K*) if the dielectric constant *ϵ*_s_ is lower than that of water (as it is in methanol-water solvents and in pure methanol). Actually the *pK* for acids of this charge type has been found to be from 3 to 5 units higher in methanol than in water.^2^

If the equilibrium is of the charge type A^+^B^0^ (for example, A^+^= anilinium cation, B^0^= uncharged aniline; *z*_B_=0) and *r*_H_ is about equal to *r*_A_, however, the electrostatic treatment alone would lead one to expect *pK* to be almost unaffected by changes in the dielectric constant of the solvent. It is unlikely that *r*_A_ will often be less than *r*_H_, and therefore an increase in *pK* would be more easily explained on electrostatic grounds than would a decrease. In actuality, there are several instances where *pK* for acids of this charge type has been found to be nearly the same in pure methanol as in water [3]. There is evidence, however, that the *pK* of amines decreases when methanol is added to the water solvent; it passes through a minimum at a solvent composition in the region 60 to 80 wt percent methanol and rises again at high methanol concentrations.^3^

This type of shift of the equilibrium constant of an isoelectric process with composition of the solvent is of interest for its implications concerning the relative basicities of water and methanol in the mixed solvents, about which there is as yet no general agreement. For example, the conductance of hydrochloric acid in methanol-water mixtures appears to indicate that protons are bound more tightly to water molecules than to methanol [8], and equilibrium studies have led to the same result [9]. On the other hand, Feakins and Watson [10] have concluded that the standard free energies of transfer of the halogen acids from water to the mixed solvent (10 or 43.12 wt percent methanol) indicate that the proton is in a state of lower energy in the methanol-water solvents than in pure water. Similar conclusions have been drawn by Wells [11] from the behavior of *p*-nitroaniline in solutions of strong acids in methanol and isopropanol.

The dissociation constants of two substituted anilinium acids, namely *o*-chloroanilinium ion and *m*-nitroanilinium ion, have been determined in solutions of hydrochloric acid by spectrophotometric methods in methanol-water solvents of composition varying from 0 to 93.7 wt percent methanol (*m*-nitroanilinium) or 99.9 wt percent (*o*-chloroanilinium). The *pK* of both acids has been found to decrease as the solvent is enriched with methanol up to a composition of about 70 mole percent, where it passes through a minimum and rises rapidly as the last 10 percent of water is removed from the mixed solvent. The contrasting behavior of an acid of the charge type A^0^B^−^ has also been demonstrated by parallel measurements on 4-chloro-2,6-dinitrophenol at three methanol concentrations between 0 and 33.5 wt percent.

## 2. Experimental Methods

### 2.1. Materials

*o*-Chloroaniline was purified by distillation at reduced pressure (about 16 mm Hg) and was stored in the dark. *m*-Nitroaniline was recrystallized once from methanol, and 4-chloro-2,6-dinitrophenol was crystallized three times from methanol.

The spectro grade methanol used in the study of *o*-chloroaniline was distilled and the first fraction collected was discarded. The lot used in the measurements was found by Karl Fischer titration to contain 0.09 percent water. This grade of methanol was of sufficiently high quality that it was used without distillation in the study of the other two indicators.

Hydrochloric acid, purified by distillation, was used to prepare the solutions in solvents containing at least 10 wt percent of water, but diluted reagent grade acid was used without further purification to obtain the limiting absorptions of the acidic forms of the three substances studied.

For solvents of high methanol concentration, a reagent solution was prepared by passing gaseous hydrogen chloride (generated by adding concentrated aqueous hydrochloric acid dropwise to concentrated sulfuric acid) into methanol. The hydrochloric acid solutions were standardized by titration with a standard aqueous solution of sodium hydroxide. At the highest methanol concentrations, the limiting absorption of the alkaline form of the indicator was measured in a solution of sodium methoxide formed by dissolving a piece of clean sodium metal in methanol. The concentration of this solution was determined by titration with a standard aqueous solution of hydrochloric acid.

### 2.2. Procedures

The several components of the solutions were weighed so that the weight composition of the solvent and the molality of each constituent could be calculated. The spectral absorbance of the solutions containing *o*-chloroaniline was measured by a Beckman Model DU spectrophotometer the cell compartment of which was controlled at 25 °C. In general, the absorbance was determined at 10 to 12 different wavelengths extending from 10 m*μ* below the maximum of the absorption band to 10 m*μ* above. The value of *α*, the fraction of the indicator present in the base form, was computed from each measurement, and the results were averaged.

An Optica single-beam spectrophotometer was employed for the measurements with *m*-nitroaniline and 4-chloro-2,6-dinitrophenol. The room in which the instrument was located was maintained at a temperature close to 25 °C. The measurements were made at the wavelength at which the absorptions of the base forms (the free aniline and the phenolate ion) were at a maximum in water. These wavelengths were 360 m*μ* and 450 *mμ*, respectively. The solvents used in the study of *m*-nitroanilinium ion varied in composition from pure water to 93.7 wt percent methanol, while the dissociation of 4-chloro-2,6-dinitrophenol was studied in pure water, 16.2 percent methanol, and 33.5 percent methanol.

## 3. Results. Calculation of pK

The spectrophotometric data for *o*-chloroanilinium ion are summarized in table 1. The values of $\log\frac{\alpha }{1-\alpha }$ for mixtures of *o*-chloroaniline and hydrochloric acid (third column) are the averages of about 10 measurements made at wavelengths near the maximum of the absorption band. This quantity is log [(*D*−*D*_1_)/(*D*_2_−*D*)], where *D*_1_, *D*_2_, and *D* are respectively the absorbance of the acid form of the indicator molecule, of the base form, and of the solution of the partially transformed indicator, determined in the same or identical cells at identical total concentrations of the absorbing substance. The molality of hydrochloric acid is given in the first column, and the logarithm of the molality of the hydrogen ion at equilibrium (taking into account the acid-base reaction with the aniline) is given in the second column.

The values of *p*(_s_*K*)′ for *o*-chloroanilinium ion were calculated by the equation

$$p{\left({\;}_{s}K\right)}^{\prime }\equiv p\left({\;}_{s}K\right)+\log_{s}\left(\frac{\gamma_{H}\gamma_{H}}{\gamma_{{\text{HB}}^{+}}}\right)=-\log m_{H}-\log\frac{\alpha }{1-\alpha },$$ (8)

where B represents the free aniline base and *γ_i_* is the activity coefficient on the molal basis, here referred to the standard state in the methanolic solvent s.

Activity coefficient terms of this form and charge type have been found to vary in proportion to the ionic strength in 33.4 wt percent methanol, as they do in water [12]. Hence, *p*(_s_*K*) is the intercept at *I*=0 of the best straight line through the several values of *p*(_s_*K*)′. The values of *p*(_s_*K*) are given at the end of each section of the table. The estimates of the standard deviation of a point from the extrapolation line are marked “S.D.”

Similar but less extensive data were obtained for the acids *m*-nitroanilinium ion and 4-chloro-2,6-dinitrophenol. The total concentration of the indicator (sum of the concentrations of the acid and base forms) was 4×10^−4^*M* for *m*-nitroamlinium ion and 1×10^−4^ for 4-chloro-2,6-dinitrophenol. The molar extinction coefficient of *m*-nitroaniline (the base form) remained constant at 1,390 up to a methanol concentration of 87.8 wt percent but rose to 1,410 in 93.7 percent methanol. The corresponding extinction coefficient for the chloro-dinitrophenolate ion was 6,880 in water and increased to 6,920 and 7,070 in 16.2 and 33.5 wt percent methanol, respectively.

The values of *p*(_s_*K*)′ for *m*-nitroanilinium ion were calculated by eq (8). For 4-chloro-2,6-dinitrophenol they were calculated by the equation

$$p{\left({\;}_{s}\;K\right)}^{\prime }\equiv p\left({\;}_{s}\;K\right)+bm=-\log m_{H}-\log\frac{\alpha }{1-\alpha }+\frac{A\sqrt{m}}{1+1.5\left(78.30/\epsilon_{s}\right)\sqrt{m}}$$ (9)

where the activity coefficient term *γ*_H_*γ*_B–_/*γ*_HB_ has been expressed by a form of the Debye-Hückel equation; m is the molality of hydrochloric acid and *A* is the Debye-Hückel slope at the dielectric constant (*ϵ*_s_) of the methanolic solvent [13]. For *m*-nitroanilinium ion in solvents of methanolic content from 0 to 42.6 wt percent, there was no apparent trend of *p*(_s_*K*)′ with m (that is, *b* was zero). At the higher concentrations of methanol, however, the thermodynamic *p*(_s_*K*) was determined by extrapolation of *p*(_s_*K)'* to *m*=0. Likewise the values of *p*(_s_*K*)′ for 4-chloro-2,6-dinitrophenol did not appear, within the error of the experiments, to change with *m*. The *p*(_s_*K*) for this acid was therefore obtained by averaging the separate values of p(_s_*K*)′.

The results for these two acids are given in table 2, together with the estimates of the standard deviations (S.D.) of a single point. The *pK* values found in pure water are in excellent agreement with earlier results, for example *m*-nitroanilinium, 2.46i at 25° (Biggs and Robinson [14]), and 4-chloro-2,6-dinitrophenol, 2.97 at 25° (Bates and Schwarzenbach [15]). The *p*(_s_*K*) values in the methanolic solvents were plotted as a function of *N*_2_, the mole fraction of methanol, and the values given in table 3 were interpolated for round values of the weight percentage of methanol. The values of *p*(*K*)−*p*(_w_*K*) at round values of the mole fraction (*N*_2_) of methanol are given in table 4.

## 4. Discussion

**Figure 1:**
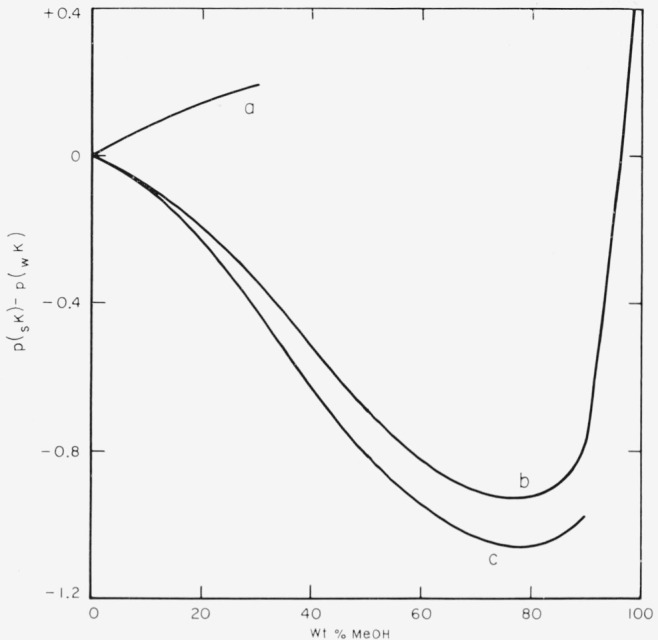
Change of *pK* for three acids plotted as a f unction of the weight percent of methanol in the solvent. a, 4-chloro-2,6-dinitrophenol; b, *o*-chloroaniliniimi ion; c, *m*-nitroanilinium ion. The ordinate represents the difference between the *pK* in the methanolic solvent, *p*(_s_*K*), and that in pure water *p*(_w_*K*).

The medium effect of methanol on the dissociation of the three acids studied is easily seen in figure 1, where *p*(_s_*K*) − *p*(_w_*K*) is plotted as a function of the weight percentage of methanol. The values of *p*(_s_*R*) for the two anilinium ions decrease as the solvent is enriched with methanol and pass through a minimum at a solvent composition of about 70 mole percent methanol. This composition is close to that at which the conductance of hydrochloric acid in methanol-water solvents is also at a minimum [4].

It is apparent that the behavior of the dissociation constants of the two anilinium ions (charge type A^+^B^0^) as the composition of the solvent is changed is not consistent with the simple Born electrostatic treatment of eq (7), which is based on the work of charging spherical ions in a dielectric continuum. This equation, together with reasonable values of the ionic radii, cannot explain either the decrease in *pK* or the minimum observed at about 70 mole percent methanol. On the contrary, the increase in *p*(_s_*K*) for 4-chloro-2,6-dinitrophenol (charge type A^0^B^−^) as the dielectric constant of the medium decreases is in qualitative agreement with the predictions of eq (7).

It is therefore of interest to compare the observed increase of *p*(_s_*K*) for 4-chloro-2,6-dinitrophenol (table 4) on addition of methanol to the solvent with the predictions of the Born electrostatic treatment, eq (7). On substitution of appropriate values of *N,e, R, T, z*_A_, *z*_B_, and *ϵ*_w_, we obtain for 25 °C

$$p\left({\;}_{s}K\right)-p\left({\;}_{w}K\right)=121.6\left(\frac{1}{\epsilon_{s}}-0.0128\right)\left(\frac{1}{r_{B}}+\frac{1}{r_{H}}\right)$$ (10)

where *r_i_* is in angstrom units. The spherical radii in solution are, of course, not known. That for hydrogen ion can be estimated to be 0.86 Å by interpolation in Pauling’s table [16] of crystal radii for a univalent cation of mass 19. The radius of the phenolate ion is certainly greater than that of the hydrogen ion. From molecular models, a figure of 2.0 Å would seem to be reasonable.

When these values of $r_{H^{+}}$ and $r_{B^{-}}$ are substituted in eq (10), the predicted increase in *pK* on passing from the water solvent to a methanolic solvent of *N*_2_=0.3 is 0.82 unit as compared with 0.25 unit observed. Even if the radius of the phenolate ion were taken as infinity, the predicted value of *p*(_s_*K*)−*p*(_w_*K*) would be reduced only to 0.58 unit. Values for the radii which are consistent with the observed result are equally unreasonable, for example $r_{H^{+}}=r_{B^{-}}=4.0\;Å$ and $r_{H^{+}}=2.4Å$, $r_{B^{-}}=11\;Å$. Furthermore, a decrease in *pK* for the two anilines (charge type A^+^B^0^) as the solvent is enriched with methanol (lowering the dielectric constant) can be accounted for on electrostatic grounds only if the radii of the anilinium ions are smaller than that of the hydrogen ion, as shown by eq (7).

In actuality, dissociation constants on the molal scale in solvents of different compositions are not strictly comparable unless an allowance is made for the variation in the total number of moles in 1000 g of solvent when the composition of the solvent changes. In other words, unit activity of solvent (in terms of which the values of *pK* are expressed) is 55.51 moles/1000 g in the pure aqueous medium and (55.51 − 24.30 *N*_2_) moles/1000 g in mixed methanol-water solvents. For a comparison at the same number of solvent molecules as are present in 1000 g of pure water, therefore, one should reduce the values of *p*(_s_*K*) in methanolic solvents by log (1 − 0.438 *N*_2_), that is, by amounts varying from 0.02 unit at *N*_2_=0.1 to 0.25 unit at *N*_2_=1. This effect amounts to only 0.06 unit at *N*_2_=0.3, and so cannot explain the wide disparity between the observed medium effect on the dissociation of 4-chloro-2,6-dinitrophenol and that predicted by the Born equation. Furthermore, this correction actually enhances the decrease found for the *pK* of the two anilinium ions.

It seems evident, therefore, that nonelectrostatic factors exert a considerable influence on the dissociation of acids of both charge types as the composition of the methanol-water solvent changes. The dissociation equilibrium $A+\text{SH}⇌B+{\text{SH}}_{2}^{+}$ (where SH represents the amphiprotic solvent) is shifted toward the right by an amount greater than can be accounted for by electrostatic considerations alone. This observation applies with equal force to acid-base pairs of the charge types A^0^B^−^ and A^+^B^0^. It is tempting to conclude that the total basicity of the medium is increased by the addition of methanol. However, it is impossible to say whether this increase stems from a higher intrinsic basicity of methanol molecides than of water molecules or whether the addition of methanol provides a larger number of basic water molecules through a breakdown of the complex water structure [17], although the latter view seems the more reasonable.

It is clear that the effect of a decreased dielectric constant will tend to shift an equilibrium of the charge type A^0^B^−^,

$$\text{HA}+\text{SH}⇌{\text{SH}}_{2}^{+}+A^{-}$$

toward the left, increasing the value of *p*(_s_*K*_HA_*).* If, in addition, the solvent basicity decreases as methanol is added this increase in *pK* will be accentuated, whereas it will be lessened if the solvent basicity increases. When the acid-base equilibrium is of the charge type A+B^0^,

$${\text{HA}}^{+}+\text{SH}⇌{\text{SH}}_{2}^{+}+A$$

the effect of a change in basicity will be the same as before (that is, a decrease of solvent basicity will tend to shift the equilibrium to the left, increasing *pK*) but the effect of a decreased dielectric constant will be much smaller and probably will tend also to increase *pK*, for the radius of the ion HA^+^ will usually exceed that of ${\text{SH}}_{2}^{+}$.

Although the Born equation alone quite evidently is unable to account successfully for the change in the free energy of dissociation for acids of the two charge types on addition of methanol, there remains the possibility that this equation furnishes reasonably accurate values of the electrostatic term, $\Delta G_{\text{el}}^{0}$. However, a nonelectrostatic term, $\Delta G_{\text{non}}^{0}$, characteristic largely of the basicity^4^ of the medium, would have to be added to it:

$$\Delta G_{\text{diss}}^{0}=\Delta G_{\text{el}}^{0}+\Delta G_{\text{non}}^{0}.$$ (11)

In the same way, we may regard the observed change of *pK*, (Δ*pK*)_ohs_, to be the sum of two terms (Δ*pK*)_el_ and (Δ*pK*)_non_. The validity of this view can be tested by determining whether the data for all three of the acids studied will furnish consistent values of (Δ*pK*)_non_ for the methanolic solvents when reasonable values for the radii of the ions are used.

The values of (Δ*pK*)_obs_, that is *p*(_s_*K*)−*p*(_w_*K*), were corrected as described above for mass law effects resulting from the different numbers of solvent, molecules present. The dielectric constants were taken from the paper of Albright and Gosting [13],^5^ and *r*_H_+ was taken to be 0.86 A. When (Δ*pK*)_el_ was calculated by eq (7) with reasonable radii for the substituted anilinium and phenolate ions and subtracted from (Δ*pK*)_ohs_, the values of (Δ*pK*)_non_ given in table 5 were obtained.

The values found by Shedlovsky and Kay [4] for the dissociation constant of acetic acid in methanol-water solvents have been similarly treated and the results are given in the last column of table 5. The *pK* values of Shedlovsky and Kay were based on the volume concentration scale and were accordingly corrected to the molal scale for comparison with the data reported here. Although the agreement in the values of (Δ*pK*)_non_ derived from the four sets of data is satisfactory, it was necessary to choose a very small radius (0.63 Å) for the acetate ion in order to bring the results into essential harmony.

Tabagua [20] has shown that addition of methanol to aqueous solutions of trichloroacetic acid, benzoic acid, salicylic acid, or diethylbarbituric acid produces an increment in *pK* comparable with that found for acetic acid. The broad distinction between the effect of methanol on the dissociation of an acid of the A^0^B^−^ type and on an acid of the A^+^B^0^ type is further supported by the work of Everett and Wynne-Jones [21], who determined the dissociation constants of ammonia and methylamine in 60 wt percent methanol over a range of temperatures.

Nevertheless, the values of (Δ*pK*)_non_ for acids of different charge types agree approximately in spite of the restrictions of the Born treatment and the arbitrary choice of ionic radii. The validity of separating medium effects on *pK* values into two parts: (a) an electrostatic part characteristic of the ion sizes, the dielectric constant of the medium, and the charge type of the acid-base equilibrium, and (b) a nonelectrostatic part characteristic of the chemical properties of the solvent, thus seems to be supported by these results. In our opinion, this approach may provide a means of assigning acid-base reaction parameters to solvent mixtures and warrants further study. The fact that (Δ*pK*)_non_ has a negative sign would suggest that the effective basicity of most methanol-water mixtures is greater than that of water itself.

Although the minimum in *p*(_s_*K*) for the two cation acids at a mole fraction of methanol of about 0.7 is probably attributable to the change in the total basicity of solvent molecules, it seemed desirable to ascertain whether the formation of ion pairs in the solvents of lowest dielectric constant might explain this phenomenon. The estimate was made at 87.8 and 100 wt percent methanol using values of 0.23 and 0.059, respectively, for the dissociation constant of hydrochloric acid in these solvents [4]. The effect of ion pairing is to make *m*_H_ in eq (8) less than the stoichiometric molality of hydrocholric acid. In the extreme case, the reduction was found to be sufficient to change *p*(_s_*K*) by only 0.03 unit in the two solvents of highest methanol content. The general conclusions are thus unaffected by ignoring the ion-pair equilibrium.

It is well known that solutions of the strong acids appear stronger in methanol and ethanol than in pure water itself,^6^ and the same seems to be true for methanol-water solvents and ethanol-water solvents as well [24, 25]. An increase in acidity or “proton activity” might signify a *decrease* in solvent basicity, for the binding between solvent molecules and protons seems to be weakened. As Hammett has shown [26], this is not necessarily the case. The basicity of the solvent can be expressed in terms of K_SH_, the acidity constant of the solvent. Lf leveling of monobasic strong acids (molality m) is complete in the solvent SH,

$$a_{H}=mK_{\text{SH}}\frac{\gamma_{\text{SH}}{\;}_{2}^{+}}{\gamma_{\text{SH}}}.$$ (12)

As the solvent basicity increases, *K*_SH_ decreases, but a lowering of the dielectric constant increases the activity coefficient term sharply. From the data of Gutbezahl and Grunwald [24], $\log\left(\gamma_{{\text{BH}}^{+}}/\gamma_{B}\right)$ for *B* = ammonia or aniline is estimated to be about 0.8 in 30 mole percent ethanol. Thus it is quite possible that the increase in the activity coefficient ratio may outweigh the decrease in *K*_SH_. The increase in the acidity of the solution is thus not necessarily inconsistent with the indicator results which seem to suggest that addition of methanol to water initially increases the total basicity of the medium. After a mole fraction of about 0.8 methanol is reached, the basicity of the solvent mixture begins to decrease and may fall to a value less than that of water itself.

## Figures and Tables

**Table 1:** Determination of *p*(_s_*K*) for *o*-chloroanilinium ion in water and in methanol-water solvents by the spectrophotometric method at 25 °C

*m*_HCl_	−log *m*_H_	$\log\frac{\alpha }{1-\alpha }$	*p*(_s_*K*)′

Water; indicator 3.46×10^−4^*M*			

0.000404	3.445	0.825	2.620
.001616	2.828	.215	2.613
.002522	2.629	.004	2.625
.003533	2.477	−0.139	2.616
.006062	2.235	−.373	2.608
.01010	2.008	−.624	2.632
			*p*(_s_K) =2.615
			S.D.= 0.008

8.0 wt percent MeOH; indicator 3.72×10^−4^*M*			

0.001041	3.025	0.469	2.556
.002083	2.715	.168	2.547
.003124	2.533	−0.027	2.560
.005208	2.304	−.254	2.558
.01041	1.995	−.567	2.562
			*p*(_s_K) =2.552
			S.D.= 0.005

16.2 wt percent MeOH; indicator 3.72×10^−4^*M*, 3.43×10^−4^*M*			

0.002112	2.703	0.237	2.466
.003167	2.523	.055	2.468
.004223	2.395	−0.073	2.468
.005278	2.296	−.175	2.471
.01056	1.989	−.498	2.486
			*p*(_s_K)=2.459
			S.D.=0.001

24.6 wt percent MeOH ; indicator 3.72×10^−4^*M*, 3.43× 10^−4^*M*			

0.001070	3.003	0.631	2.372
.002138	2.693	.342	2.351
.003209	2.514	.151	2.363
.004280	2.387	.025	2.362
.005344	2.288	−0.079	2.367
.01070	1.982	−.405	2.387
			*p*(_s_K)=2.355
			S.D.=0.008

33.4 wt percent MeOH; indicator 3.43×10^−4^*M*			

0.002173	2.683	0.458	2.225
.003256	2.504	.285	2.219
.004348	2.377	.159	2.218
.005432	2.279	.057	2.222
.008694	2.072	−0.143	2.215
.01086	1.974	−.240	2.214
.02176	1.671	−.545	2.216
			*p*(_s_K)=2.221
			S.D.=0.003

42.4 wt percent MeOH; indicator 3.43×10^−4^*M*			

0.002211	2.670	0.633	2.037
.003315	2.493	.447	2.046
.004419	2.367	.321	2.046
.005525	2.269	.220	2.049
.01105	1.965	−0.081	2.046
.02211	1.661	−.398	2.059
			*p*(_s_K)=2.041
			S.D.= 0.003

52.0 wt percent MeOH; indicator 3.43×10^−4^*M*			

0.001128	2.960	1.064	1.896
.002254	2.658	.764	1.894
.003386	2.481	.578	1.903
.004507	2.355	.464	1.891
.005645	2.258	.365	1.893
.02250	1.651	−0.250	1.901
			*p*(_s_K)=1.895
			S D.=0.004

62.3 wt percent MeOH; indicator 3.43×10^−4^*M*			

0.002313	2.645	0.869	1.776
.005790	2.243	.484	1.759
.008095	2.098	.333	1.765
.01158	1.952	.182	1.770
.01388	1.863	.097	1.767
.01737	1.766	−.005	1.771
.02315	1.640	−.128	1.768
.03473	1.463	−.300	1.763
			*p*(_s_*K*) =1.769
			S.D.=0.005

73.4 wt percent MeOH; indicator 3.72×10^−4^*M*			

0.01192	1.939	0.241	1.698
.01792	1.753	.059	1.694
.02386	1.627	−.069	1.696
.02729	1.568	−.137	1.705
			*p*(_s_*K*) =1.691
			S.D.=0.004

82.1 wt percent MeOH; indicator 3.72×10^−4^*M*			

0.002456	2.619	0.940	1.679
.006144	2.220	.535	1.685
.01223	1.918	.219	1.699
.01814	1.747	.038	1.709
.02376	1.629	−.079	1.708
.03605	1.452	−.252	1.704
			*P*(_s_*K*) = 1.685
			S.D.= 0.008

87.8 wt percent MeOH; indicator 3.82×10^−4^*M*			

0.002481	2.617	0.800	1.817
.003105	2.520	.715	1.805
.003730	2.440	.633	1.807
.004969	2.315	.507	1.808
.006216	2.217	.420	1.797
.01242	1.914	.104	1.810
.01693	1.778	−.040	1.818
.02412	1.623	−.191	1.814
.04810	1.321	−.519	1.840
			*p*(_s_*K*) =1.804
			S.D.=0.006

99.9 wt percent MeOH; indicator 3.82×10^−4^*M*			

0.000426	3.570	0.228	3.342
.000788	3.281	−.073	3.354
.001420	2.967	−.377	3.344
.001607	2.904	−.459	3.363
.002979	2.591	−.747	3.338
.008764	2.081	−1.345	3.426
			*p*(_s_K) =3.336
			S.D.=0.014

**Table 2:** *p*(_s_*K*) for *m*-nitroanilinium ion and 4-chloro-2,6-dinitrovhenol in methanol-water solvents at 25 °C

Wt % MeOH	0	8.0	16.2	24.6	33.4	42.6	59.0	80.0	87.8	93.7

*m*-Nitroaniliniuin ion										

*p*(_s_*K*)	2.460	2.402	2.267	2.132	1.944	1.790	1.505	1.393	1.428	1.679
S.D	0.003	0.004	0.011	0.012	0.005	0.012	0.004	0.010	0.010	0.011

4-Chloro-2,6-dinitrophenol										

*p*(_s_*K*)	2.969	……	3.089	……	a3.177					
S.D	0.007	……	0.004	……	0.004					

a In 33.5 wt % methanol.

**Table 3:** Interpolated values of *p*(_s_*K*) for *o*-chloroanilinium ion, *m*-nitroanilinium ion, and 4-chloro-2,6-dinitrophenol in methanol-water solvents at 25 °C

Wt % Me OH	*o*-Chloroanilinium	*m*-Nitroanilinium	4-Chloro-2,6-dinitrophenol
			
0	2.61_5_	2.46_0_	2.96_9_
10	2.53	2.37	3.05
20	2.42	2.22	3.11
30	2.27	2.03	3.16
40	2.09	1.83	……………
50	1.92	1.63	……………
60	1.79	1.50	……………
70	1.71	1.43	……………
80	1.68	1.39	……………
90	1.92	1.48	……………
99.9	3.34	……………	……………

**Table 4:** Medium effects on *pK* for o-chlor oanilinium ion, m-nitroanilinium ion, and 4-chloro-2,6-dinitrophenol in methanol-water solvents at 25 °C

*N*_2_		*p*(_s_*K*)−*p*(_w_*K*) for	
		
	*o*-Chloroanilinium	*m*-Nitroanilinium	4-Chloro-2,6-dinitrophenol
			
0	0	0	0
0.1	−0.15	−0.17	+0.12
0.2	−.35	−.45	+.19
0.3	−.58	−.69	+.25
0.4	−.75	−.90	…………………
0.5	−.86	−1.00	…………………
0.6	−.92	−1.05	…………………
0.7	−.93	−1.07	…………………
0.8	−.81	−1.03	…………………
0.9	−.42	−0.74	…………………
0.999	+0.75	…………………	…………………

**Table 5:** Values of the nonelectrostatic contribution to the medium effect *p*(_s_*K*) − *p*(_w_*K*) in methanol-water solvents

Mole fraction methanol N_2_	(Δ*pK*)_non_ from measurements of—			
	*o*-Chloroanilinium ion^a^	*w*-Nitroanilinium ion^b^	4-Chloro-2,6-dinitrophenol^c^	Acetic acid^d^
				
0.1	−0.3	−0.3	−0.2	−0.2
.2	−0.6	−0.6	−0.4	−0.4
.3	−1.0	−1.0	−0.7	−0.7
.4	−1.3	−1.3	…………	−1.0
.5	−1.5	−1.5	…………	−1.3
.6	−1.8	−1.7	…………	−1.6
.7	−2.0	−1.9	…………	−1.9
.8	−2.0	−2.0	…………	−2.2
.9	−1.9	−1.9	…………	−2.2
1.0	−1.0	…………	…………	−1.5

a $r_{A^{+}}=2Å$.

b $r_{A^{+}}=1.5Å$.

c $r_{B^{-}}=1.5Å$.

d $r_{{\text{Ac}}^{-}}=0.63Å$.

## Footnotes

1 Figures in brackets indicate the literature references at the end of this paper.

2 See, for example, references 3 and 4.

3 See, for example, references 5, 6, and 7

4 The term *nonelectrostatic* is used for want of a satisfactory one-word designation for basicity effects and interactions involving the solvent in general. The electrostatic term connotes the ion-ion interaction of long-range coulombic forces as well as ion-charging effects; it is this interaction which is the subject of Bjerrum’s description of dibasic acids [18] and its subsequent refinement by Kirkwood and Westheimer [19]. Interactions other than those of the ion-ion type, designated here as “basicity effect” or “nonelectrostatic term” may also be electrical in origin, as in the interaction of an ion with the dipole of a solvent molecule.

5 See also reference 4.

6 See, for example, references 22 and 23.
